# Progress in Ophthalmology

**Published:** 1898-02-05

**Authors:** 


### PROGRESS IN OPHTHALMOLOGY.

(*Continued from page* 294.)

**Treatment of Catarrhal Ophthalmia.**—Sydney Stephenson[11] states that catarrhal ophthalmia is the result of a specific bacillus, which he describes as varying in size from 0·75 $\mu$. to 1 $\mu$; they resemble rather closely the bacilli of mouse septicæmia, but are smaller, and have rounded instead of angular ends; they never show any sign of segmentation or sporulation. With regard to the treatment, he says that nitrate of silver is the sovereign remedy for catarrhal ophthalmia. The 2 per cent. solution in general use can hardly be improved upon, and should be brushed over the everted conjunctiva, which has been first carefully freed from secretion. The acuter symptoms seldom withstand more than three or four applications. Added to this as a wash he recommends one-eighth of a grain of corrosive sublimate to an ounce of distilled water, with the addition of one grain of ammonium chloride. This collyrium warmed may be used from three to eight times a day. When the acuter symptoms have passed silver is to be replaced by some less stimulating eye wash, such as two grains of lapis divinus and 30 minims of landanum to ounce of distilled water. Zinci sulph. or alum lotion (1-3 grs. to ℥) succeeds well in some cases. He also mentions having had good results from use of the "yellow lotion of the Austrian Pharmacopœia."

In conclusion, he says: Let me earnestly insist on the inadvisability of allowing patients with catarrhal ophthalmia to be associated in the same wards with those affected with the purulent disease or with trachoma.

**Cycling as Cause of Eye Worry.**—Dr. Mirovitch[12] points out the various ways in which cycling causes eye-worry. First as to the position of the body—and his remarks apply more to the racing cyclist than to the ordinary rider—the body being bent forward, the head is not held in the erect position; therefore, to see in front, the superior recti and the oblique muscles are in use all the time, and the eyeballs are drawn upwards and inwards, so that the upper part of the cornea is hidden. The vision takes place through the lower part of the cornea, the part least practised in distant vision. This habit, he says, must produce modification in the movements of the eyeballs (superior or spasmodic strabismus), as well as in the refraction of the eye. As well as this there is the congestion of head and eyes caused by stooping. Cyclists who ride at a great rate, too, suffer much from the strong and constant pressure of the air; especially the internal angles suffer, as these angles may be considered as the summits of the cavities formed by the edge of the nose and by the plane of the eye.

**Asepsis v. Antisepsis**—In the *Clinical Journal* Dr. Ludwig Bach[13] discusses the various advantages of asepsis over antisepsis. He points out the impossibility of keeping the conjunctival sac sterile, and therefore says antiseptics are useless, or almost useless, and act as irritants. He deplores the too rigorous measures which are often practised before eye operations, such as continuous flushings and moppings of conjunctiva and lids several days before operation, with perhaps the application of what is misnamed "an antiseptic bandage"; this he points out as being especially harmful, inasmuch as it prevents winking, and hinders the departure of the germs that are always and must always be present. In short, Bach advocates strict cleanliness, with the perfect sterilisation of the instruments, and that any instrument which is to penetrate the eye must not be allowed to touch the lid margins or dip into the fluid in the conjunctival sac. With regard to the after treatment he maintains that germs which are to do harm must be pressed in between the lips of the wound, as might occur in rubbing the eye, and that the germs passing over the surface of the wound are fairly harmless.

**Astringents in the Treatment of Eye Diseases**—The *Therapeutic Gazette*, in quoting a paper of Edgar A. Browne, says, in relation to the abuses of astringents in eye diseases, that of the principal astringents in use the sulphate of copper is the best. Against alum he has an objection. As to chloride of zinc he says, "though there are better, it may still be regarded as an excellent remedy," but has the disadvantage of being extremely irritating. Nitrate of silver cannot be done without—the conjunctiva will stand much but the cornea very little of it, and if the cornea be abraded it will give rise to white opacities.

**Loss of Vision of Circulatory Origin.**—J. B. Barrett,[15] of Melbourne, mentions several cases of sudden loss of vision of circulatory origin, and says that temporary loss of vision may be produced by (1) spasm of the retinal arteries; (2) complete embolism of the retinal artery; (3) partial embolism of the retinal artery, probably combined with spasm; and (4) narrowing of the retinal arteries from arterial disease and temporary heart failure. Several of the causes may co-operate with one another. Sudden permanent blindness may be produced by (1) spasm of the retinal vessels; (2) complete embolism; (3) partial embolism with temporary spasm of the arteries; and (4) thrombosis with or without spasm.

**Formalin in Eye Diseases.**—Strozeminski,[16] of Wilna, says that he has employed formalin in many diseases of the eye, but not with very brilliant results. In most, if not all, of the conjunctival affections, it is useless; also in blepharitis, when used as an ointment (1 in 400), it is not only useless, but produces extreme irritation. In ulcers of the cornea, with or without hypopyon, formalin c̄ atropin had a favourable influence, and in serpiginous ulcer formalin rapidly induced a cure. From this it would appear that it is fatal only to certain forms of micro-organisms, others being immune to it.

**Holocaine and Eucain.**—In the *Medical Chronicle* of June, 1897,[17] there is a very eulogistic notice of holocaine as compared with cocaine—inasmuch as it is much more powerful in its effects, a one per cent. sol. causing anæsthesia of the cornea in something under two minutes; furthermore, it does not cause contraction of the vessels or drying of the corneal epithelium as cocaine does. As well as having these advantages it is in itself antiseptic and so does not require sterilisation—one disadvantage is its highly poisonous nature, which prevents its use in subconjunctival injections. With regard to eucain it is said to be better than cocaine as the anæsthesia lasts longer, with no dilatation of the pupil or paralysis of accommodation. In iritis it was not so good as cocaine, as the ischæmic action of the latter drug was absent.

**Gonn. Ophthal. treated with Argonin.**—In the *American Medico-Surgical Bulletin* for August 25th, 1897, a case is reported by F. T. Smith[18] of gonorrhœal ophthalmia, treated by application of argonin. After the usual treatment of boric acid, bichloride of mercury, and nitrate of silver, which had very little effect on the discharge, a microscopic examination of the discharge showed many gonococci before, but after the argonin they disappeared rapidly; in the discharges that escaped there were none; and in a sample taken from inside of the lid they were very very few. From this the author concludes that (1) argonin can be used safely for the eye; (2) that it is less irritating than nitrate of silver; and from its wonderful effect on the discharge, and on the development of gonococci, it appears to be the ideal remedy in gonorrhœal ophthalmia.

**Kindergarten and Eyesight.**—In the *New York Med.*

*Journal*, Dr. Wood,[19] in an article on the Kindergarten system, deprecates the employment of young children's eyes for near work, inasmuch as he thinks it tends to render them myopic; some of the work being very fine and requiring very close attention, while the children are, as all young children are, hypermetropes. He advocates the testing of the children's sight before they are allowed to do any work, and says, "No child with uncorrected or incorrigible defects should be allowed to use his eyes for any kind of close work before he is eight or nine years of age, lest worse things befall"; and goes on to say, "my plea, therefore, is for work that one connects with a real garden—a genuine child's garden. Plenty of air and sunshine, a minimum of instruction, with a maximum of natural enjoyment, these I would have in every kindergarten."

[11] Lancet, June 5, 1897, page 1,531. [12] Bulletin de la Société Francaise d'Hygiene, Mar. 11, 1897, No. 1,068. [13] Clinical Joural, Aug., 1897. [14] Therapeutic Gazette, May 15, 1897, page 339. [15] Intercolonial Medical Journal of Australasia, Vol. II., No. 3. [16] Medical Chronicle, June, 1897, page 234. [17] Medical Chronicle, Jan., 1897, page 195. [18] American Med. and Surg. Bulletin, Aug. 25, 1897, p. 754. [19] New York Medical Journal, July 17, 1897, p. 74.

